# A reprogrammed genetic code consisting of 32 distinct amino acids

**DOI:** 10.1093/nar/gkag140

**Published:** 2026-02-18

**Authors:** Takayuki Katoh, Hiroaki Suga

**Affiliations:** Department of Chemistry, Graduate School of Science, The University of Tokyo, 7-3-1 Hongo, Bunkyo-ku, Tokyo 113-0033, Japan; Department of Chemistry, Graduate School of Science, The University of Tokyo, 7-3-1 Hongo, Bunkyo-ku, Tokyo 113-0033, Japan

## Abstract

Sense codon reassignment enables ribosomal incorporation of nonproteinogenic amino acids (npAAs) at any of the 61 sense codons. Because npAAs replace proteinogenic amino acids (pAAs), the total number of available building blocks usually remains limited to 20. To overcome this, we previously introduced “artificial codon box division”, where four-codon boxes (e.g. Val GUN) are split into distinct sets (e.g. GUY and GUG) using *in vitro* transcribed transfer RNAs (tRNAs) lacking nucleotide modifications. This allows two different amino acids—a pAA and an npAA—to be assigned within the same original box. While we previously demonstrated this by incorporating 23 amino acids, low incorporation efficiency hindered further expansion. Here, we applied our engineered tRNAs, tRNA^Pro1E2^ and tRNA^iniP^, to the codon box division framework and optimized translation conditions to facilitate multiple npAA incorporations. Consequently, we successfully expanded the genetic code to 32 amino acids, incorporating 11 elongator npAAs and 1 initiator npAA while maintaining all 20 pAAs. Notably, these npAAs include therapeutically significant monomers such as β-amino, d-amino, and *N*-methyl amino acids, as well as an initiator *N*-chloroacetyl-d-tyrosine for peptide macrocyclization. This platform offers vast potential for generating diverse macrocyclic peptide libraries with unique chemical entities for drug discovery.

## Graphical Abstract

**Graphical Abstract:**
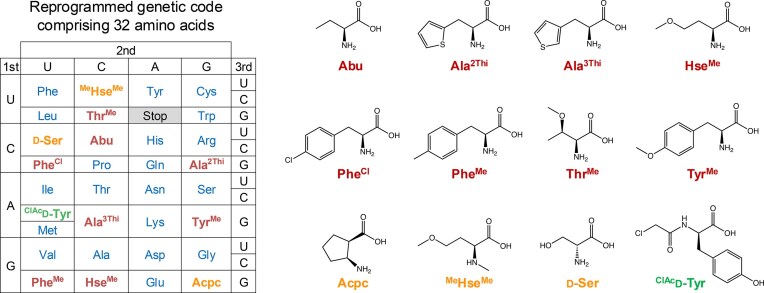

## Introduction

**Figure 1.**
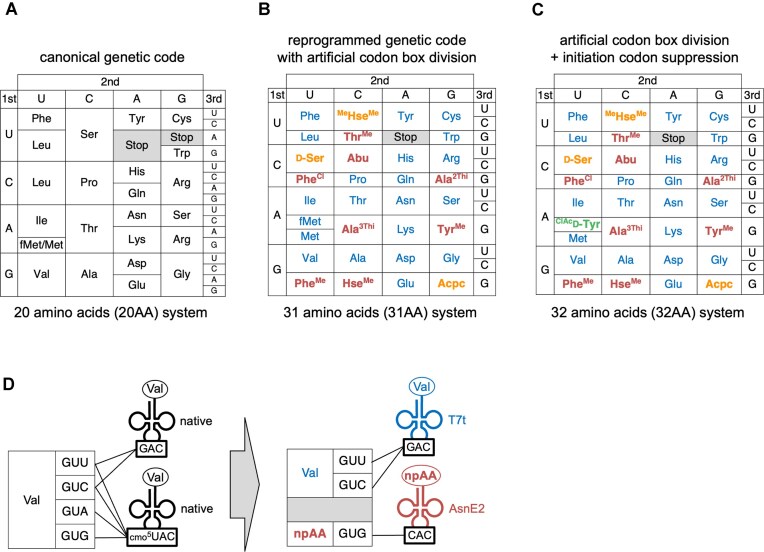
Reprogramming genetic code by means of sense codon reassignment and codon box division. **(A–C)** Canonical and reprogrammed genetic codes. Canonical genetic code [panel (A), 20AA system], reprogrammed genetic codes comprised of 31 amino acids [panel (B), 31AA system], and 32 amino acids [panel (C), 32AA system]. The 31AA system consists of the 20 pAAs and 11 elongator npAAs. In the 32AA system, one initiator npAA was added to the 31AA system. **(D)** Division of the Val GUN codon box. The canonical translation system uses two native *Escherichia coli* tRNA^Val^s bearing GAC and cmo^5^UAC anticodons for decoding, while T7-transcribed (T7t) tRNA^Val^_GAC_ and tRNA^AsnE2^_CAC_ were used for introducing Val and npAA, respectively, in the codon box division.

In nature, 20 proteinogenic amino acids (pAAs) are used for the translation of peptides and proteins (Fig. 1A, canonical genetic code); however, the development of genetic code manipulation methodologies has enabled the introduction of diverse nonproteinogenic amino acids (npAAs). For instance, the stop codon suppression method repurposes one or more of the three stop codons (UAG, UAA, and UGA) to incorporate npAAs [1]. However, since at least one stop codon must be retained for translation termination, this approach typically allows for the addition of only one or two npAAs, limiting the total alphabet to 22 amino acids (20 pAAs + 2 npAAs). Alternatively, quadruplet codons (programmed frameshift codons) can be generated from “rare codons” to expand the genetic code up to 24 amino acids [2, 3]. Nevertheless, because rare codons are scarce and engineering additional quadruplet codons without sacrificing decoding fidelity is arduous, this number (24 amino acids) likely represents the practical limit of this approach.

In contrast, sense codon reassignment utilizes any of the 61 sense codons for npAA incorporation [4]. In this framework, the pAA typically assigned to a reprogrammed codon is replaced by an npAA. As the 20 pAAs are redundantly assigned to the 61 sense codons, it is theoretically possible to introduce up to 61 different amino acids by completely breaking codon degeneracy. However, this degeneracy is strictly controlled by transfer RNA (tRNA) molecules, which limits for such a complete breaking. For example, the *E. coli* translation system decodes the Val GUN codon box using two isoacceptor tRNAs with GAC and cmo^5^UAC anticodons (cmo^5^U stands for carboxymethoxyuridine, a modified nucleotide). Due to flexible wobble base pairing at the third codon position [5], these two tRNAs cover all four Val codons; GAC anticodon decodes GUU and GUC, while cmo^5^UAC can decode all four GUN codons (Fig. 1D, left) [6]. Consequently, dividing the Val GUN box is infeasible as long as the tRNA bearing cmo^5^UAC anticodon is present. Similar restrictions apply to the Leu CUN, Ser UCN, Pro CCN, Thr ACN, and Ala GCN boxes due to modified uridines at the first anticodon position (Supplementary Fig. S1).

To overcome this hurdle, our group developed the concept of “artificial codon box division”, using *in-vitro* transcribed tRNAs lacking nucleotide modifications in a custom-made *E. coli in-vitro* translation system, referred to as Flexible *In-vitro* Translation (FIT) system [7, 8]. To decode the aforementioned Val GUN codon box, we used an *in-vitro* transcribed tRNA^Val^_GAC_ whose GAC anticodon was unmodified but yet capable of decoding half of the Val codon box (GUU and GUC). Even though the specific nucleotide modification of the native tRNA^Val^ was missing in this transcript, valyl-tRNA synthetase (ValRS) is capable of charging Val to produce Val-tRNA_GAC_. Simultaneously, we introduced an artificial orthogonal tRNA (tRNA^AsnE2^) with a CAC anticodon [9], which is not recognized by ValRS but can be aminoacylated with an npAA using flexizymes (tRNA aminoacylation ribozymes) [10, 11]. This allowed us to split the Val GUN box, assigning Val to GUU/GUC and an npAA to GUG (Fig. 1D, right; i.e. GUA codon was not utilized in this study). By applying this strategy to the Arg CGN and Gly GGN boxes, we previously expanded the genetic code to 23 amino acids (20 pAAs + 3 npAAs) [10].

While this concept could theoretically support up to 31 elongator amino acids, expanding beyond 23 proved challenging due to low incorporation efficiencies and premature peptide termination caused by peptidyl–tRNA drop-off. To reach higher numbers while maintaining all 20 pAAs, we fine-tuned the FIT system by: (i) engineering tRNAs that more efficiently recruit EF-Tu and EF-P, and (ii) optimizing translation factor concentrations. Regarding the approach of (i), we have introduced an engineered elongator tRNA, tRNA^Pro1E2^, whose T-stem and D-arm motifs were derived from tRNA^Glu^ and tRNA^Pro1^, respectively [12–15]. We also developed a new initiator tRNA, tRNA^iniP^, whose D-arm was derived from tRNA^Pro1^. Their D-arms recruit EF-P in the E-site, resulting in efficient translation using npAAs [16]. The T-stem of tRNA^Pro1E2^ promotes accommodation of aminoacyl–tRNA to the A-site by efficiently recruiting EF-Tu. Regarding the approach (ii), we optimized the concentrations of EF-Tu, EF-P, EF-G, IF2, IF3, and RRF [13, 16–18]. For d-amino acid elongation, concentrations of IF2, EF-Tu, and EF-G were adjusted to 3, 20, and 0.1 µM, respectively (note that their concentrations in the original FIT system were 0.4, 10, and 0.26 µM, respectively) [13]. As a result, we observed more than a five-fold improvement of the translation yield of a model peptide containing two consecutive d-Ala. Moreover, by adding 5 µM EF-P, two consecutive d-Ala incorporation was enhanced by another five-fold when using tRNA^Pro1E2^ [14]. Similar enhancements were achieved for β-amino acid incorporations [19]. For the initiation event, we optimized the concentrations of IF3, EF-G, EF-P, and RRF to be 15, 1, 10, and 2.5 µM, respectively (their concentrations in the original FIT system were 1.5, 0.26, 0, and 0.5 µM, respectively) [16, 18]. By using tRNA^iniP^ for incorporation of *N*-acetylproline at the N-terminus under these conditions, we observed ∼1000-fold increase of the expression level of a model peptide. Incorporation of d-amino, β-amino, and γ-amino acids at the N-terminus was also enhanced similarly under the same conditions.

In this study, we applied these engineered tRNAs and optimized conditions to achieve the goal of incorporating 12 npAAs alongside all 20 pAAs. We first established a 31-amino acid translation system (31AA system, 11 elongator npAAs + 20 pAAs; Fig. 1B). Furthermore, by reprogramming the initiator AUG codon to *N*-chloroacetyl-d-tyrosine (ClAc-d-Tyr), we enabled spontaneous macrocyclization via thioether bond formation with a downstream cysteine. This resulted in a robust 32-amino acid translation system (32AA system, Fig. 1C), providing a powerful platform for the ribosomal synthesis of highly diverse macrocyclic peptide libraries comprising 32 different chemical entities.

## Materials and methods

### *In vitro* transcription of tRNAs and flexizymes

tRNAs and flexizymes (dFx and eFx) were prepared by *in vitro* transcription using T7 RNA polymerase. Template DNAs for transcription were prepared by extension and polymerase chain reaction (PCR) using *Taq* DNA polymerase. Extension reaction was performed at 95°C for 1 min and 5 cycles of 50°C for 1 min and 72°C for 1 min in the following reaction mixture: 10 mM Tris–HCl (pH 9.0), 50 mM KCl, 0.1% (v/v) Triton X-100, 2.5 mM MgCl_2_, 250 μM dNTP mix, 60 nM *Taq* DNA polymerase, and 2 μM forward and reverse extension primers (see Supplementary Table S1 for primer sequences). PCR was performed for 15 cycles of 95°C for 40 s, 50°C for 40 s, and 72°C for 40 s in 10 mM Tris–HCl (pH 9.0), 50 mM KCl, 0.1% (v/v) Triton X-100, 2.5 mM MgCl_2_, 250 μM dNTP mix, 60 nM *Taq* DNA polymerase, 0.5 μM forward and reverse PCR primers and 1/100 vol. of the extension reaction mixture. In the reverse PCR primers for tRNA, the second nucleotide from the 5′-end was modified with 2′-*O*-methylation. The resulting DNAs were purified by phenol/chloroform extraction and ethanol precipitation.

Transcription was carried out at 37°C for overnight in 0.5–2 ml reaction mixture: 40 mM Tris–HCl (pH 8.0), 22.5 mM MgCl_2_, 1 mM dithiothreitol (DTT), 1 mM spermidine, 0.01% Triton X-100, 3.75 or 5 mM nucleoside triphosphate (NTP) mix, 5 or 0 mM guanosine monophosphate (GMP), 0.04 U/µl RNasin RNase inhibitor (Promega, M6101), 0.12 µM T7 RNA polymerase, and 0.5–2-ml-scale PCR product. Concentration of NTP mix was 3.75 or 5 mM and GMP was 5 or 0 mM for tRNA and flexizyme, respectively. The resulting RNAs were treated with RQ1 DNase (Promega) at 37°C for 30 min and purified by 8% (tRNA) or 12% (flexizyme) denaturing polyacrylamide gel electrophoresis.

### Aminoacylation of tRNAs

**Figure 2.**
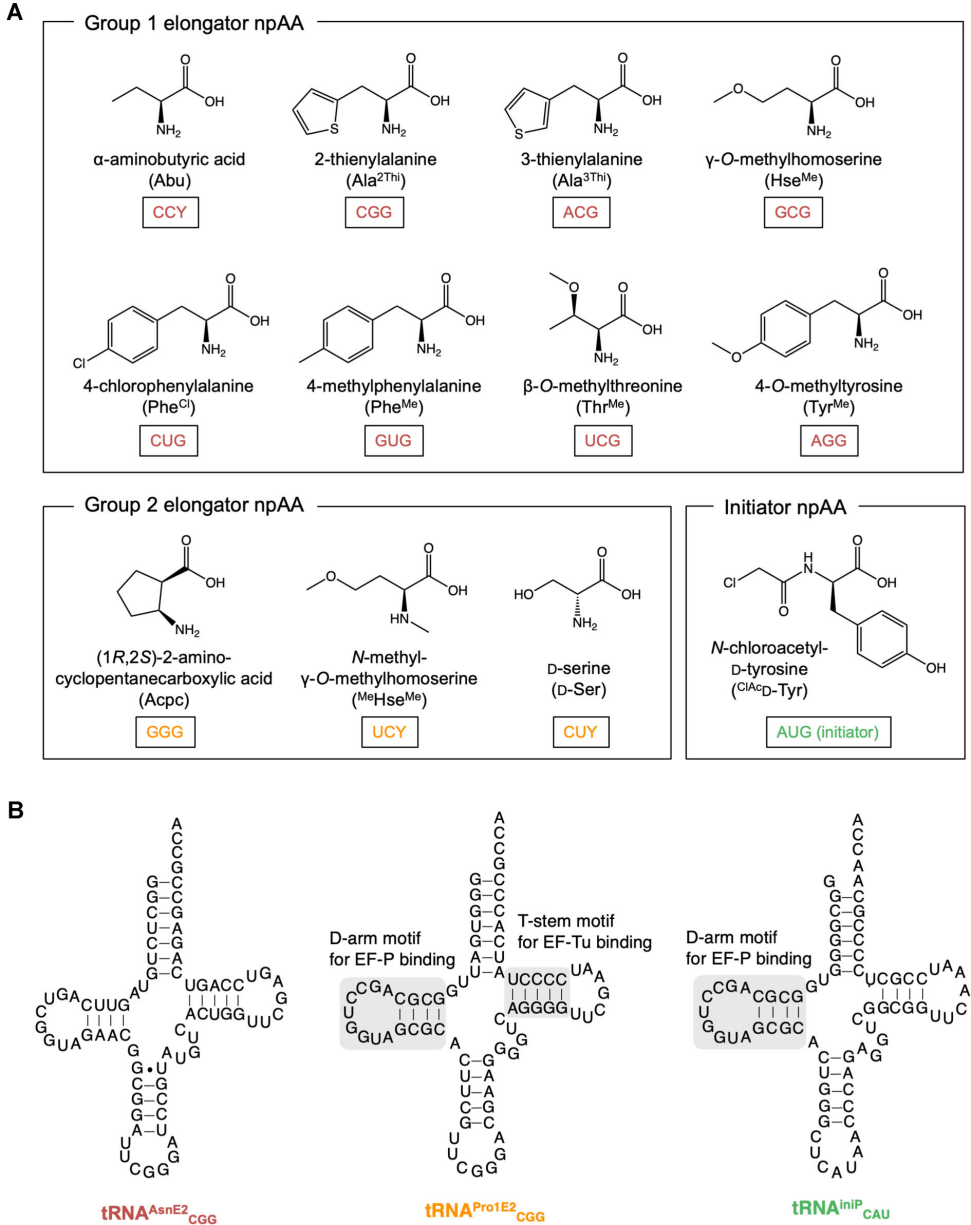
Nonproteinogenic amino acids and engineered tRNAs used in this study. **(A)** Chemical structures and abbreviations of npAAs. **(B)** Secondary structures of engineered tRNAs. The D-arm and T-stem motifs recognized by EF-P and EF-Tu are highlighted.

2-thienylalanine (Ala^2Thi^), 3-thienylalanine (Ala^3Thi^), 4-chlorophenylalanine (Phe^Cl^), 4-methylphenylalanine (Phe^Me^), 4-*O*-methyltyrosine (Tyr^Me^), and ^ClAc^d-Tyr were pre-activated as cyanomethyl ester (CME) and α-aminobutyric acid (Abu), (1*R*,2*S*)-2-aminocyclopentanecarboxylic acid (Acpc), γ-*O*-methylhomoserine (Hse^Me^), *N*-methyl-γ-*O*-methylhomoserine (^Me^Hse^Me^), d-serine (d-Ser), and β-*O*-methylthreonine (Thr^Me^) were pre-activated as 3,5-dinitrobenzyl ester (DBE) by previously reported methods (Fig. 2A) [20, 21]. Aminoacylation was performed at 0°C in 50 mM HEPES–KOH (pH 7.5) or Bicine–KOH (pH 8.7), 600 or 100 mM MgCl_2_, 20% dimethylsulfoxide, 25 μM eFx or dFx, 25 μM tRNA, and 5 mM activated amino acid. eFx and dFx were used for CME- and DBE-activated substrates, respectively. Concentration of MgCl_2_ was 600 mM for eFx and 100 mM for dFx. The reaction time was 2 h for Phe^Cl^, Phe^Me^, Tyr^Me^, and ^ClAc^d-Tyr, 4 h for Abu, Hse^Me^, and d-Ser, 6 h for ^Me^Hse^Me^ and Thr^Me^, 16 h for Acpc, Ala^2Thi^, and Ala^3Thi^. The reaction pH was 8.7 for Acpc and 7.5 for the others. The resulting aminoacyl–tRNAs were recovered by ethanol precipitation, washed twice with 70% ethanol containing 100 mM sodium acetate (pH 5.2), and dissolved in 1 mM sodium acetate (pH 5.2).

### *In vitro* translation of model peptides

**Figure 3.**
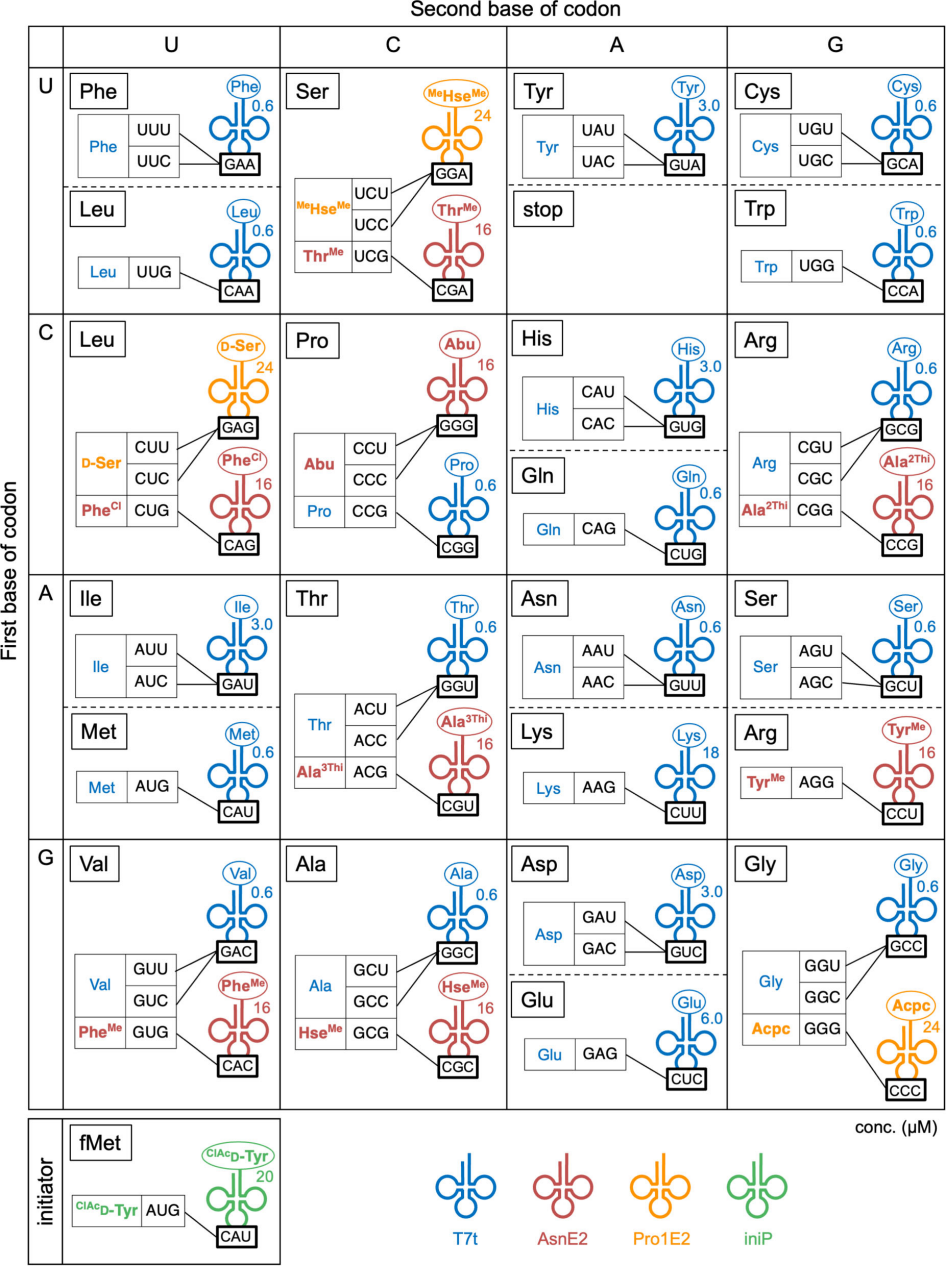
Concentrations and anticodon sequences of tRNAs used for incorporation of the 32 amino acids. Elongator npAAs introduced by tRNA^Pro1E2^ and tRNA^AsnE2^ are highlighted in orange and brown, respectively. pAAs introduced by T7t tRNA are indicated in blue. Initiator ^ClAc^d-Tyr is indicated in green.

*In vitro* translation was performed at 37°C for 30 min in a 2.5 µl FIT system: 50 mM HEPES–KOH (pH 7.6), 100 mM potassium acetate, 12.6 mM magnesium acetate, 0.1 mM 10-formyl-5,6,7,8-tetrahydrofolic acid, 2 mM ATP, 2 mM GTP, 1 mM CTP, 1 mM UTP, 20 mM creatine phosphate, 2 mM spermidine, 1 mM DTT, 0.6–18 µM T7t tRNAs, 16 µM each npAA–tRNA^AsnE2^, 24 µM each npAA–tRNA^Pro1E2^, 1.2 µM *E. coli* ribosome, 0.6 µM methionyl–tRNA formyltransferase, 2.7 µM IF1, 3 µM IF2, 1.5 µM IF3, 20 µM EF-Tu/Ts, 0.26 µM EF-G, 5 µM EF-P, 0.25 µM RF2, 0.17 µM RF3, 0.5 µM RRF, 4 µg/ml creatine kinase, 3 µg/ml myokinase, 0.1 µM inorganic pyrophosphatase, 0.1 µM nucleotide diphosphate kinase, 0.1 µM T7 RNA polymerase, 0.73 μM AlaRS, 0.03 μM ArgRS, 0.38 μM AsnRS, 0.13 μM AspRS, 0.02 μM CysRS, 0.06 μM GlnRS, 0.23 μM GluRS, 0.09 μM GlyRS, 0.02 μM HisRS, 0.40 μM IleRS, 0.04 μM LeuRS, 0.11 μM LysRS, 0.03 μM MetRS, 0.68 μM PheRS, 0.16 μM ProRS, 0.04 μM SerRS, 0.09 μM ThrRS, 0.03 μM TrpRS, 0.02 μM TyrRS, 0.02 μM ValRS, 0.5 mM each pAA mix, and 1.6 µM DNA template. See Fig. 3 for the concentrations of T7t tRNAs. Then, 5 µM tRNA^fMet^ was added when fMet was introduced at the N-terminus. For the incorporation of npAAs, 16 µM each npAA–tRNA^AsnE2^ and 24 µM each npAA–tRNA^Pro1E2^ were added to the above system; This translation system is referred to as the 31AA system (Fig. 1B). See Supplementary Table S1 for the sequences of tRNAs.

For reprogramming the initiator fMet with ^ClAc^d-Tyr, 10-formyl-5,6,7,8-tetrahydrofolic acid and tRNA^fMet^ were removed, 20 µM ^ClAc^d-Tyr-tRNA^iniP^ was added, and the concentrations of IF3 and RRF were increased to 15 and 2.5 µM, respectively (Fig. 1C, the 32AA system).

For the translation of p3-1–5_20_, the T7t tRNAs and pre-charged npAA–tRNAs were omitted and 1.5 mg/ml *E. coli* total tRNA was added; This translation system is referred to as the 20AA system (Fig. 1A).

EF-G concentration was fixed at 0.26 µM. EF-G translocates peptidyl–tRNA from the ribosome A-site to P-site but also promotes the unreacted peptidyl–tRNA drop-off and reinitiation when the elongation was slow, leading to the production of truncated peptide fragments. Therefore, in our previous study, we reduced the EF-G concentration from 0.26 to 0.1 µM for efficient npAA elongation [17]. However, in the initiation event, EF-G suppresses the drop-off-reinitiation event at the N-terminus and thus its concentration should be increased to around 1 µM to efficiently obtain full-length peptides [18]. As the final goal of this study is introduction of both initiator and elongator npAAs, we decided to keep the original concentration, 0.26 µM, which is in between 0.1 and 1 µM.

The resulting peptides were desalted by SPE C-tip (Nikkyo Technos), co-crystalized with α-cyano-4-hydroxycinnamic acid on a sample plate and analyzed by MALDI-TOF mass spectrometry (MS) in a reflector-positive mode using UltrafleXtreme (Bruker Daltonics). A peptide calibration standard II (Bruker Daltonics) was used for external mass calibration.

For quantification of peptides, translation was carried out in the presence of 0.04 mM [U-^14^C]-Glu in place of cold Glu, quenched by adding the same volume of stop solution [0.9 M Tris–HCl (pH 8.45), 8% sodium dodecyl sulphate, 30% glycerol, and 0.001% xylene cyanol], and incubated at 95°C for 2 min. Peptides were separated by 15% tricine sodium dodecyl sulphate–polyacrylamide gel electrophoresis (SDS–PAGE) and analyzed by autoradiography using a Typhoon FLA 7000 (Cytiva). The translation levels of peptides were estimated by the autoradiographic intensity relative to total [U-^14^C]-Glu in the reaction.

## Results

### Development of the 31-amino acid system

We first attempted to develop a 31-amino acid (31AA) system by introducing 11 elongator npAAs (Fig. 1B). We selected two groups of npAAs: Group 1, consisting of eight l-α-amino acids with sidechain modifications (Abu, Ala^2Thi^, Ala^3Thi^, Hse^Me^, Phe^Cl^, Phe^Me^, Thr^Me^ and Tyr^Me^; Fig. 2A); and Group 2, containing three peptide-backbone-altering npAAs (Acpc, ^Me^Hse^Me^ and d-Ser; Fig. 2A). Since the incorporation of Group 1 npAA is fairly efficient compared to Group 2, they were charged on a set of conventional tRNAs derived from tRNA^Asn^ orthogonal to *E. coli* aminoacyl–tRNA synthetases (aaRS), referred to as tRNA^AsnE2^_NNN_ (where NNN represents the anticodon), and introduced at CCY, CGG, ACG, GCG, CUG, GUG, UCG, and AGG codons, respectively (Figs 2B and 3, and Supplementary Table S1; Y = U or C). In contrast, Group 2 npAAs are evidently more challenging substrates for elongation [9, 14, 19, 22–24]; therefore, they were introduced using tRNA^Pro1E2^_NNN_ at GGG, UCY, and CUY codons (Figs 2B and 3). All npAAs were specifically charged onto the 3′-hydroxy group of their respective tRNAs via flexizyme [10]. The 20 pAAs were introduced by *in vitro*-transcribed *E. coli* tRNAs prepared by using T7 RNA polymerase (referred to as T7t tRNAs); the respective T7t tRNAs were charged with pAAs by the cognate aaRSs *in situ* within the FIT system.

The tRNA concentrations in the translation system are summarized in Fig. 3. (Note that the initiator fMet was not reprogramed in the 31AA system and was introduced by using 5 µM T7t tRNA^fMet^). Considering the lower incorporation efficiency of Group 2 npAAs, their aminoacyl–tRNA concentrations were set 1.5-fold higher (24 µM) than those of Group 1 npAA–tRNAs (16 µM). Regarding T7t tRNAs, the concentrations of tRNA^Asp^, tRNA^Glu^, tRNA^His^, tRNA^Ile^, tRNA^Lys^ and tRNA^Tyr^ were set relatively higher (3, 6, 3, 3, 18, and 3 µM, respectively) than the other T7t tRNAs (0.6 µM), as their aminoacylation/translation efficiencies were observed to be relatively low in our previous study [7]. We also increased the concentrations of IF2, EF-Tu, and EF-P to 3, 20 and 5 µM, respectively, based on our previous optimization for d- and β-amino acid incorporation [13, 14, 19].

### Verification of codon box division

**Figure 4.**
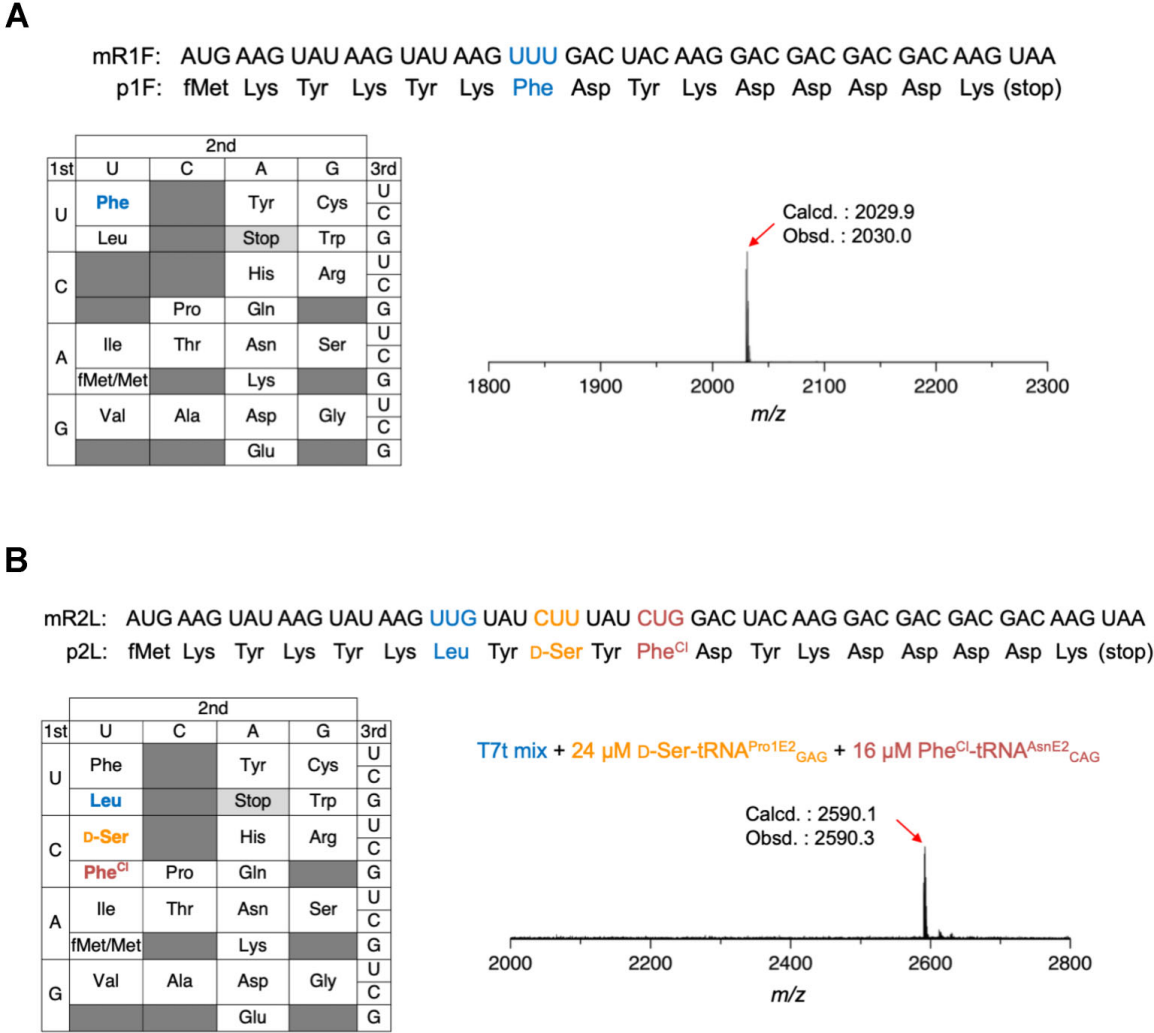
*In vitro* translation of model peptides confirming the accurate incorporation of target amino acids at designated codons. **(A)** Translation of model peptide p1F to verify Phe incorporation at the UUU codon. Shown are the sequences of mRNA mR1F and peptide p1F, the reprogrammed genetic code table used for translation, and the MALDI-TOF MS spectrum of p1F. **(B)** Translation of model peptide p2L to verify the incorporation of Leu, d-Ser, and Phe^Cl^ at the UUG, CUU, and CUG codons, respectively. Shown are the sequences of mRNA mR2L and peptide p2L, the corresponding reprogrammed codon table, and the MALDI-TOF MS spectrum of p2L. Calculated (Calcd.) and observed (Obsd.) *m/z* values are indicated.

To confirm pAA incorporation using T7t tRNAs, we first constructed a custom FIT system comprising only T7t tRNAs. No npAA–tRNAs were added, leaving the codons indicated in dark gray “empty” (Fig. 4A). Since the codon boxes for Asn, Cys, Gln, Glu, His, Ile, Met, Phe, and Trp were not divided, these pAAs were assigned solely to their original codons using T7t tRNAs. Expression of the model peptide p1F using the messenger RNA (mRNA) mR1F was confirmed by MALDI-TOF MS without any impurity peaks (Fig. 4A). Similarly, the incorporation of Asn, Cys, Gln, Glu, His, Ile, Met, and Trp at their respective codons in mR1N, mR1C, mR1Q, mR1E, mR1H, mR1I, mR1M, and mR1W was confirmed. All corresponding peptides were expressed without significant byproducts (Supplementary Fig. S2).

On the other hand, the codon boxes for Ala, Arg, Gly, Leu, Pro, Ser, Thr, and Val were divided into two or three sub-boxes to accommodate npAAs. For instance, the Leu codon box was divided into three sub-boxes: UUG (Leu), CUY (d-Ser), and CUG (Phe^Cl^) (Fig. 4B). For the translation of the model mRNA mR2L, we used a custom FIT system containing the 20 T7t tRNAs, where Leu was charged on T7t tRNA^Leu^_CAA_ by the endogenous LeuRS, while d-Ser-tRNA^Pro1E2^_GAG_ and Phe^Cl^-tRNA^AsnE2^_CAG_ were prepared via flexizymes. The mR2L has UUG, CUU, and CUG codons for incorporation of these three amino acids. In this experiment, other npAAs were not introduced and therefore their codons were left empty (dark gray boxes, Fig. 4B). MALDI-TOF MS revealed clean expression of the desired p2L, indicating that the Leu codon box was successfully divided into three codons and assigned to the three amino acids correctly (Fig. 4B). Divisions of Ala, Arg, Gly, Pro, Ser, Thr, and Val codon boxes were similarly validated using mR2A, mR2R, mR2G, mR2P, mR2S, mR2T, and mR2V, respectively (Supplementary Fig. S3). For example, the Ala codon box was divided into GCY (Ala) and GCG (Hse^Me^). (Supplementary Fig. S3A). Although a small byproduct peak was observed for mR2A/p2A due to a Hse^Me^-to-Ala misincorporation at GCG codon, these results demonstrate that eight codon boxes could be successfully partitioned to assign 11 npAAs while retaining the 8 original pAAs.

### Simultaneous incorporation of 31 amino acids

**Figure 5.**
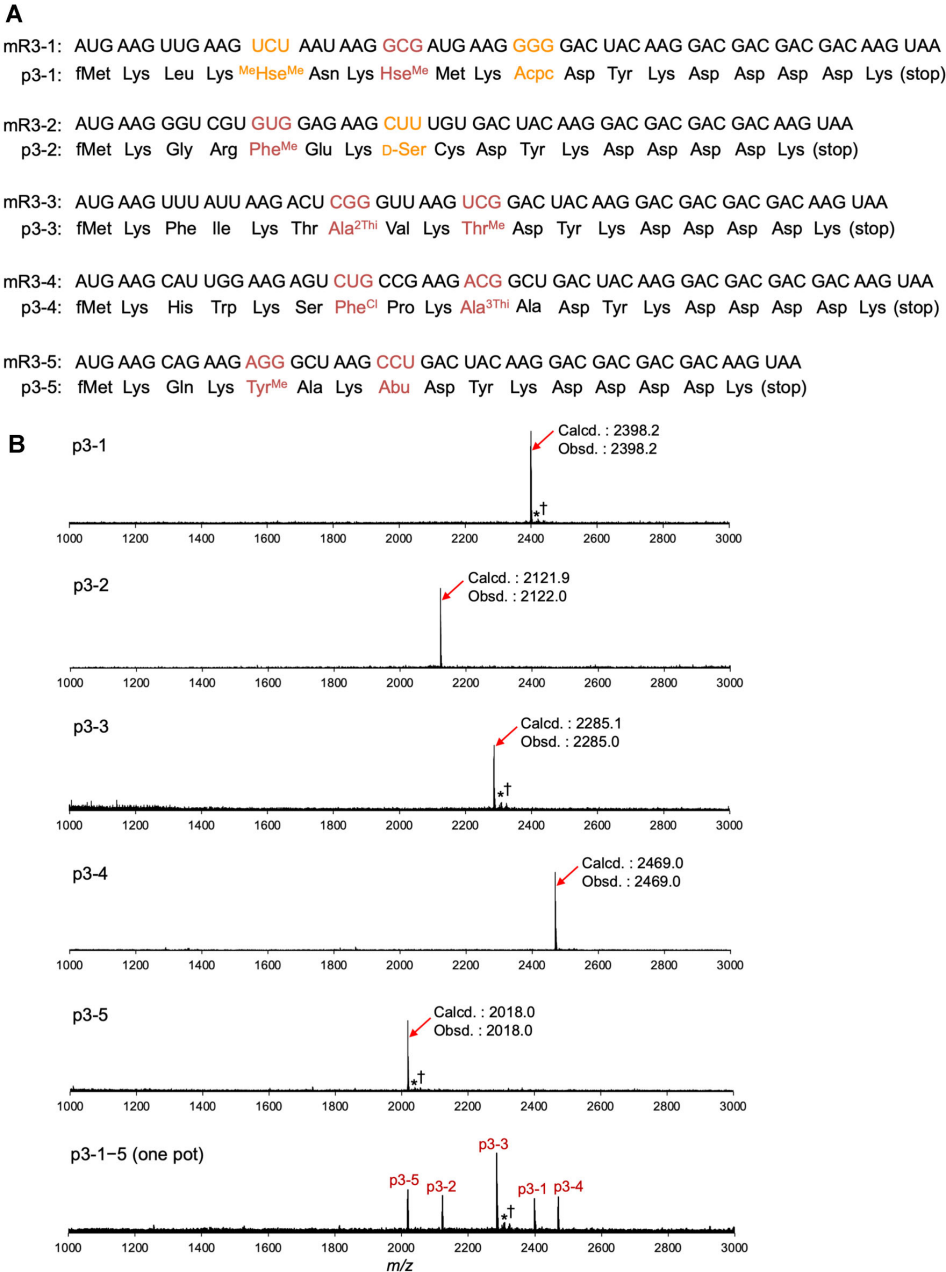
*In vitro* translation of model peptides using the 31AA system. **(A)** Sequences of the model mRNAs and peptides. **(B)** MALDI-TOF mass spectrometric analysis of the translated peptides. Calculated (Calcd.) and observed (Obsd.) *m/z* values are indicated for each peptide. “p3-1−5 (one pot)” indicates a simultaneous translation of the five peptides (p3-1, p3-2, p3-3, p3-4, and p3-5) introducing the five corresponding mRNAs (mR3-1, mR3-2, mR3-3, mR3-4, and mR3-5) into the 31AA system. * and † indicate Na^+^ and K^+^ adducts of the desired products, respectively.

Next, to demonstrate the simultaneous incorporation of 31 amino acids, we prepared five model mRNA/peptide pairs (Fig. 5A, mR3-1–5/p3-1–5). The peptide sequences were designed such that all 31 amino acids appeared at least once across the five peptides. Translation was carried out using the 31AA system that includes all the 11 elongator npAAs alongside the 20 pAAs based on the reprogrammed codon table shown in Fig. 1B. First, these five mRNAs were individually translated into the corresponding peptides and MALDI-TOF MS confirmed their expression without significant impurities (Fig. 5B, p3-1–5). We further demonstrated their simultaneous translation in a single one-pot reaction, where all five peptides were successfully expressed. To confirm translational accuracy, de novo sequencing of p3-1 was performed using MALDI-TOF/TOF MS/MS, verifying the correct sequence of the incorporated amino acids (Supplementary Fig. S4).

Finally, we evaluated the translational activity by redesigning mR3-1–5 into mR3-1–5E, adding a 4 × GAG repeat for C-terminal 4 × Glu incorporation to facilitate radiolabeling with [U-^14^C]-Glu (Supplementary Fig. S5A). Translation of these peptides was performed using the 31AA or 20AA system, yielding different peptide sequences, designated as p3-1–5E_31_ and p3-1–5E_20_, respectively. In the 20AA system, the T7t tRNAs and pre-charged npAA–tRNAs were replaced with a native *E. coli* tRNA mix. The resulting peptides were separated via Tricine SDS–PAGE and quantified by autoradiography (Supplementary Fig. S5B and C). Quantification revealed that p3-1E_31_, p3-2E_31_, p3-3E_31_, p3-4E_31_, and p3-5E_31_ translated by the 31AA system yielded 0.08, 0.16, 0.29, 0.13, and 0.21 µM, respectively, while the levels of p3-1E_20_, p3-2E_20_, p3-3E_20_, p3-4E_20_, and p3-5E_20_ using the 20AA system were 0.37, 0.61, 0.53, 0.49, and 0.67 µM, respectively. Notably, p3-1E_31_, which contains three npAAs (including two backbone-altering ones), exhibited the lowest yield, highlighting the inherent challenge in translating such npAA-rich sequences. The relative translation levels of the 31AA system ranged from 0.21 to 0.55 compared to the 20AA system, indicating an approximately two-to-five-fold reduction in efficiency. This decrease is likely attributable to the lack of nucleotide modifications in the T7t tRNAs and the lower translation efficiencies of npAAs relative to pAAs.

### The 32-amino acid system capable of expressing various macrocycles

Finally, the initiator AUG codon was also reprogrammed to introduce ^ClAc^d-Tyr (Fig. 1C) at the N-terminus, establishing a genetic code consisting of 32 amino acids (32AA system). To reprogram the initiation codon, we omitted T7t tRNA^fMet^_CAU_ and 10-formyl-5,6,7,8-tetrahydrofolic acid (the substrate for methionyl–tRNA formyltransferase required for *N*-formylation of Met-tRNA^fMet^_CAU_). Instead, ^ClAc^d-Tyr-tRNA^iniP^_CAU_ was prepared using flexizyme and added to the 31AA system. Additionally, the concentrations of IF3 and RRF were adjusted to 15 and 2.5 µM, respectively, based on our previous optimization for initiator npAA incorporation [16, 18].

**Figure 6.**
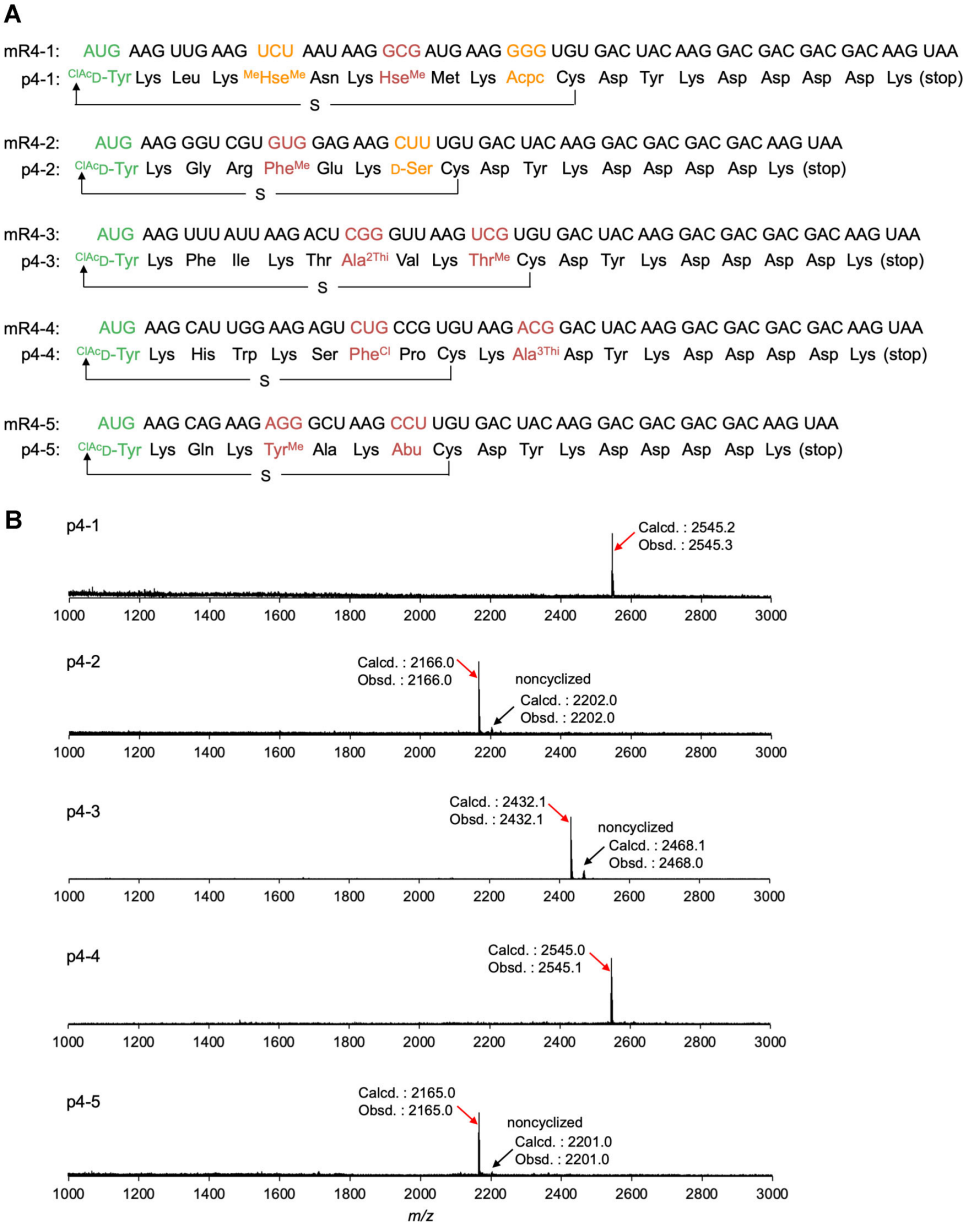
*In vitro* translation of model macrocyclic peptides using the 32AA system. **(A)** Sequences of the model mRNAs and peptides. A thioether bond was formed between the initiator ^ClAc^d-Tyr and the downstream Cys, resulting in macrocyclization of the peptides. **(B)** MALDI-TOF mass spectrometric analysis of the translated peptides. Calculated (Calcd.) and observed (Obsd.) *m/z* values are indicated for each peptide.

We designed five model mRNA/peptide pairs such that all 32 amino acids, including the initiator ^ClAc^d-Tyr, appear at least once across the five peptides (Fig. 6A, mR4-1–5/p4-1–5). Each peptide contains a Cys residue at a downstream position, whose side-chain sulfhydryl group spontaneously reacts with the N-terminal chloroacetyl group of ^ClAc^d-Tyr to form a thioether bond, thereby yielding a macrocyclic scaffold. Translation was performed according to the reprogrammed codon table shown in Fig. 1C, and the products were analyzed by MALDI-TOF MS (Fig. 6B). As expected, the peaks corresponding to the desired macrocyclic peptides were observed as the major products for every mRNA template. Although small peaks representing noncyclized byproducts were detected for p4-2, p4-3, and p4-5, these peaks indicate that while translation was completed correctly, the subsequent cyclization reaction was incomplete. Most importantly, no significant misincorporation was observed in any of the peptides. Based on these results, we concluded that 12 npAAs and 20 pAAs—a total of 32 different amino acids—were successfully encoded within the genetic code of our custom FIT system.

## Discussion

In this study, we successfully broke the degeneracy of eight codon boxes (Leu, Val, Ser, Pro, Thr, Ala, Arg, and Gly) to add 11 elongator npAAs to the codon table without sacrificing any of the 20 pAAs, establishing what we termed the 31AA system. Furthermore, by reprogramming the initiator fMet with ^ClAc^d-Tyr, we developed a 32AA system capable of expressing thioether-macrocyclic peptides. These systems drastically expand both the sequence and chemical diversity of translatable peptides. For instance, for a 20-mer peptide, the theoretical sequence diversity of the 31AA system is ∼6000-fold greater than that of the canonical 20AA system (31^20^ versus 20^20^).

While the native translation system is limited to the 20 pAAs (all l-α-amino acids) with restricted sidechain variations, our systems incorporate not only the 20 pAAs but also sidechain- and backbone-altered npAAs, including β-amino, d-α-amino, and *N*-methyl-α-amino acids. This expansion enables the exploration of vastly larger chemical and conformational spaces through *in vitro* display platforms, such as the RaPID system [8], for *de novo* discovery of bioactive peptides. Although the latter group of npAAs (backbone-altered) are generally less efficient substrates for ribosomal translation, we successfully installed four such amino acids—Acpc, d-Ser, ^ClAc^d-Tyr, and ^Me^Hse^Me^—simultaneously into the 32AA system by promoting their incorporation using tRNA^Pro1E2^ and tRNA^iniP^ in conjunction with EF-P (Fig. 1B and C).

In this work, we focused on the expression of relatively short peptides (∼20 residues), as we relied on MALDI-TOF MS for detection, a technique whose sensitivity drops significantly at *m/z* > 3000. Given the relatively lower translation efficiencies of the 31AA and 32AA systems compared to the 20AA system, synthesizing longer peptides with yields sufficient for MALDI-TOF MS remains challenging. Therefore, further enhancement of the translational activity of these systems will be necessary to enable the efficient translation of longer peptides and proteins in the future.

The division of a codon box into four independent codons is technically challenging due to wobble base-pairing between the third codon base and the first anticodon base when using the wild-type *E. coli* ribosome. Consequently, a codon box is typically divided into only two sub-boxes, NNU/C and NNG, while the NNA codon remains unutilized to avoid crosstalk. Interestingly, McFreely *et al.* recently reported that NNA and NNG could be further discriminated using T7t tRNAs in combination with a hyper-accurate ribosome mutant, mS12 [25]. In their study, the Leu UUA and UUG codons were independently decoded by two T7t tRNAs bearing UAA and CAA anticodons, respectively, to assign two distinct npAAs. Similarly, the Leu CUA and CUG codons were decoded by T7t tRNAs with UAG and CAG anticodons. While decoding with the wild-type ribosome suffered from promiscuity, the use of the mS12 ribosome improved decoding accuracy. As a result, they divided the Leu codon box (UUR + CUN) into five and the Val GUN codon box into two, assigning five npAAs while retaining Leu and Val, thereby encoding a total of 25 amino acids.

If this strategy is applicable to the division of other NNA/NNG codons, the number of available amino acids could potentially increase by up to 14 additional kinds. Thus, the next challenge in this field would be the simultaneous introduction of 20 pAAs and 26 npAAs, reaching a total of 46 different amino acids (= 32 + 14). Although such an endeavor remains a formidable task, we envision that our engineered tRNAs, tRNA^Pro1E2^ and tRNA^iniP^, used under the optimized translation conditions may facilitate the development of such an expanded FIT system in the future.

The 31AA and 32AA systems are highly compatible with the RaPID system for the *de novo* discovery of bioactive peptides [8, 26]. The enhanced structural and functional diversity of the peptide libraries generated by these systems is expected to facilitate the discovery of potent ligands against various disease-related targets. It is well established that β-amino acids often induce unique turn or helical motifs [27–30]. In particular, cyclic β^2,3^-amino acids, such as Acpc, possess strong folding propensities due to their constrained cyclic structures, which can lead to high binding affinity, enhanced proteolytic stability, and improved membrane permeability [23, 31–35]. The introduction of d-amino acids further contributes to proteolytic resistance [36–41], while *N*-methylamino acids enhance both the metabolic stability and membrane permeability of peptides [42–46]. Therefore, these npAAs represent highly attractive building blocks for peptide-based drug development. Furthermore, we have previously reported that other backbone-altering substrates, such as γ-amino acid [47, 48], α-hydrazino acid [49], α-aminoxy acid [49], and α-hydroxy acid [50], are also compatible with the FIT system. Utilizing these diverse substrates will further enhance the structural and functional variations of the resulting peptides and proteins. The synergistic effect of increased sequence diversity and the incorporation of backbone-altering npAAs will enable the exploration of unprecedented chemical space for the *de novo* discovery of bioactive peptides.

## Supplementary Material

gkag140_Supplemental_Files

## Data Availability

All data supporting this study are available in the main figures and online supplementary materials.
