# Supplementary material for: A reprogrammed genetic code consisting of 32 distinct amino acids
Source: Nucleic Acids Res. 2026 Feb 18;54(4):gkag140. doi: 10.1093/nar/gkag140 (PMC12914324; doi:10.1093/nar/gkag140)
Supplement: gkag140_Supplemental_Files [file gkag140_supplemental_files.zip › 260118_32aa-system_SI.pdf]

Supplementary Information for:

A reprogrammed genetic code consisting of 32 distinct amino acids

Takayuki Katoh[1]* and Hiroaki Suga[1]

[1]*Department of Chemistry, Graduate School of Science, The University of Tokyo, 7-3-1 Hongo, Bunkyo-ku, Tokyo 113-0033, Japan*

*Corresponding author: katoh@chem.s.u-tokyo.ac.jp

Table of Contents

**Title**	**Page**
Supplementary Fig. 1: Codon-anticodon interactions and nucleotide modifications in *E. coli* tRNAs.	3
Supplementary Fig. 2: Translation of model peptides to confirm accurate incorporation of pAAs at designated codons.	4
Supplementary Fig. 3: Translation of model peptides to confirm accurate codon box division.	7
Supplementary Fig. 4: *De novo* sequencing of peptide p3-1 by MALDI-TOF/TOF MS/MS.	10
Supplementary Fig. 5: Quantification of peptide translation level by radiolabeling.	11

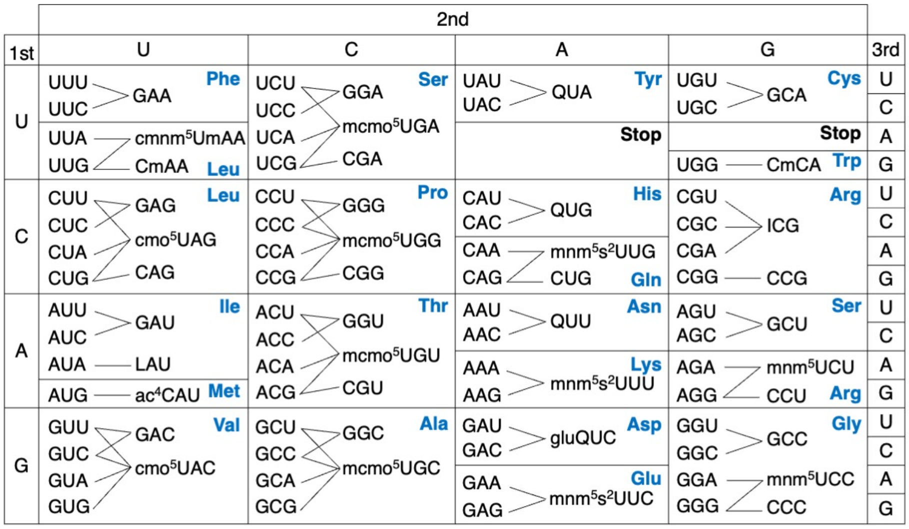

Supplementary Figure 1. Codon-anticodon interactions and nucleotide modifications in *E. coli* tRNAs. Codon and anticodon sequences are shown at the left and the right, respectively. ac^{4}C: N^4-acetylcytidine, Cm: 2′-O-methylcytidine, cmnm^{5}Um: 5-carboxymethylaminomethyl-2'-*O*-methyluridine, cmo^{5}U: 5-carboxymethoxyuridine, gluQ: glutamyl-queuosine, I: inosine, mcmo^{5}U: 5-methoxycarbonylmethoxyuridine, mnm^{5}U: 5-methylaminomethyluridine, mnm^{5}s^{2}U: 5-methylaminomethyl-2-thiouridine, Q: queuosine

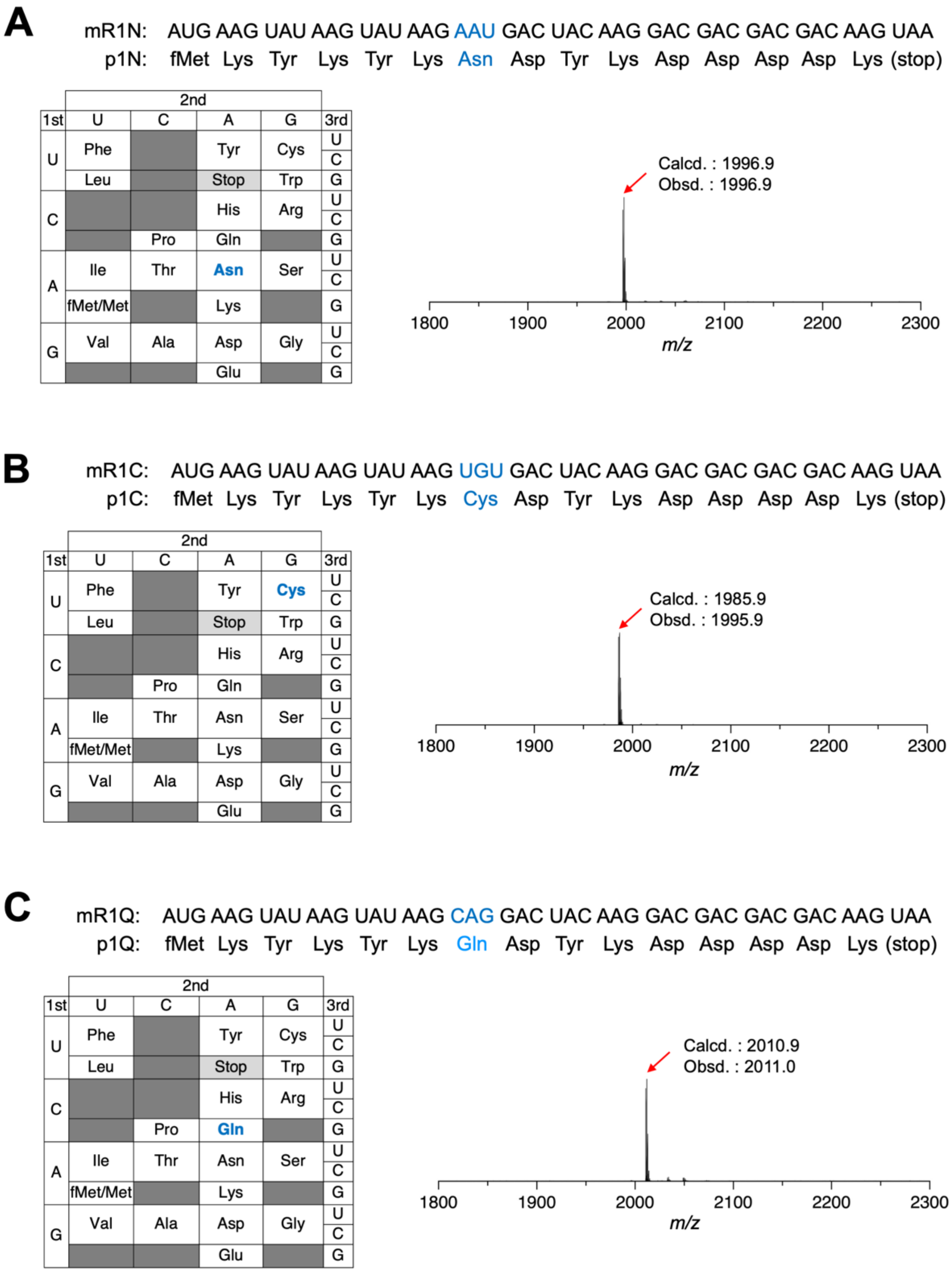

Supplementary Figure 2. Translation of model peptides confirming the accurate incorporation of pAAs at designated codons. (A−H) Presented are the sequences of the model mRNAs and peptides, the reprogrammed genetic code table used for translation, and the corresponding MALDI-TOF MS spectra of the synthesized peptides. Calculated (Calcd.) and observed (Obsd.) *m/z* values are indicated for each peptide.

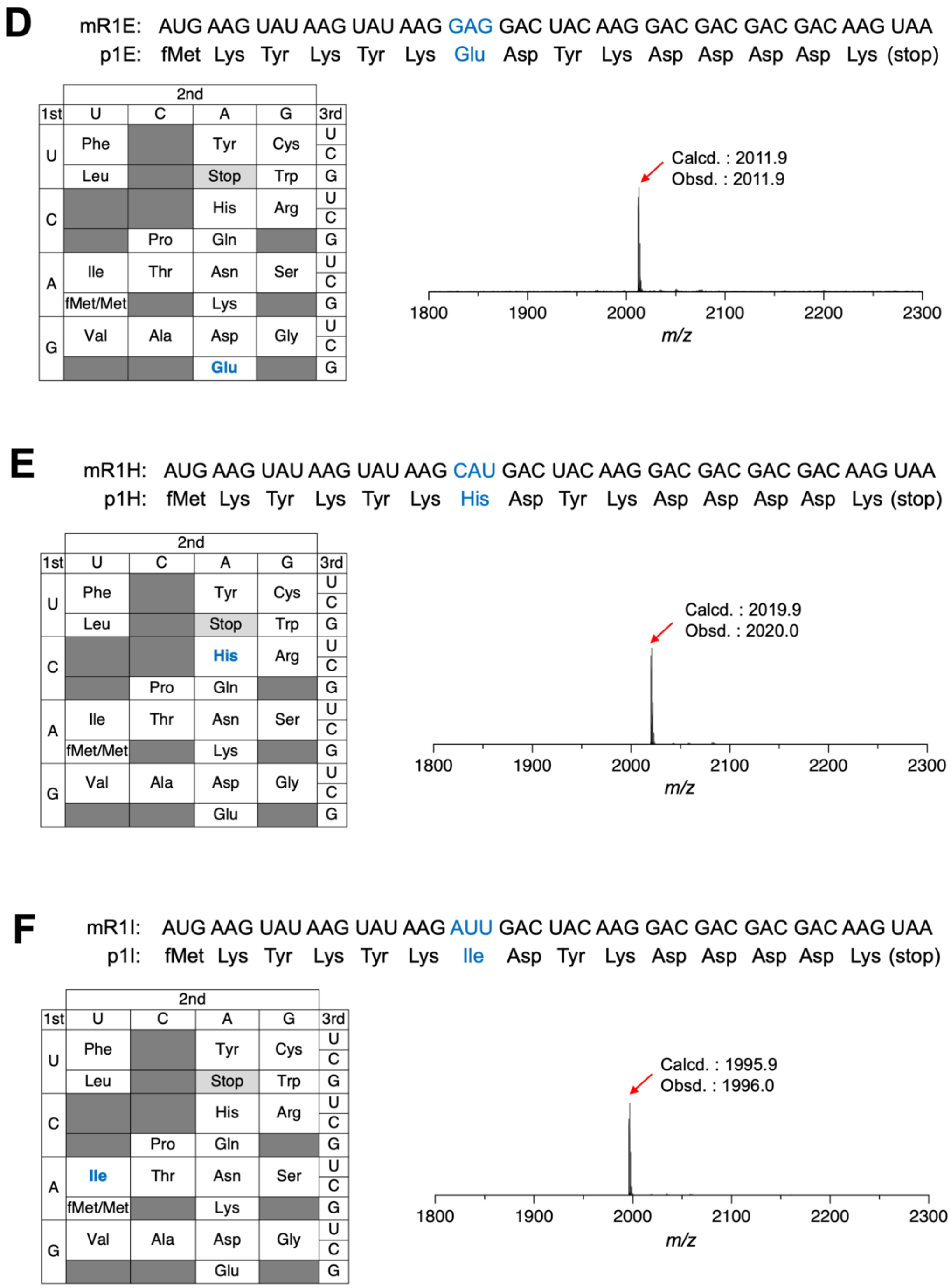

Supplementary Figure 2 continued.

G mR1M: AUG AAG UAU AAG UAU AAG AUG GAC UAC AAG GAC GAC GAC GAC AAG UAA
p1M: fMet Lys Tyr Lys Tyr Lys Met Asp Tyr Lys Asp Asp Asp Asp Lys (stop)

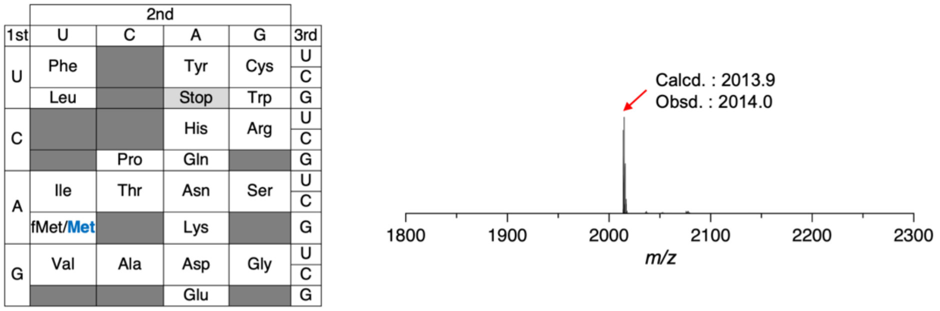

H mR1W: AUG AAG UAU AAG UAU AAG UGG GAC UAC AAG GAC GAC GAC GAC AAG UAA
p1W: fMet Lys Tyr Lys Tyr Lys Trp Asp Tyr Lys Asp Asp Asp Asp Lys (stop)

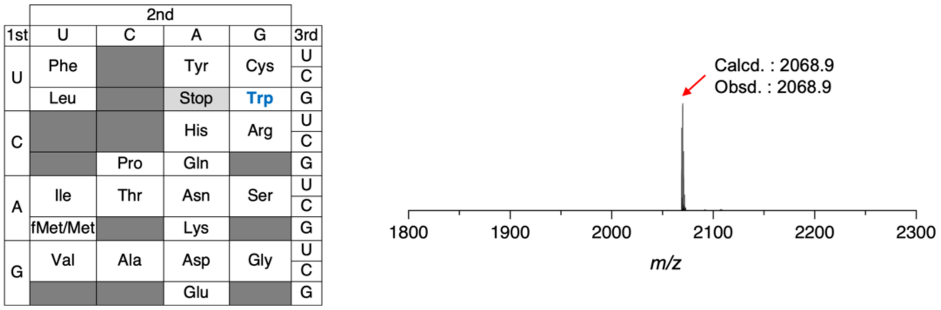

Supplementary Figure 2 continued.

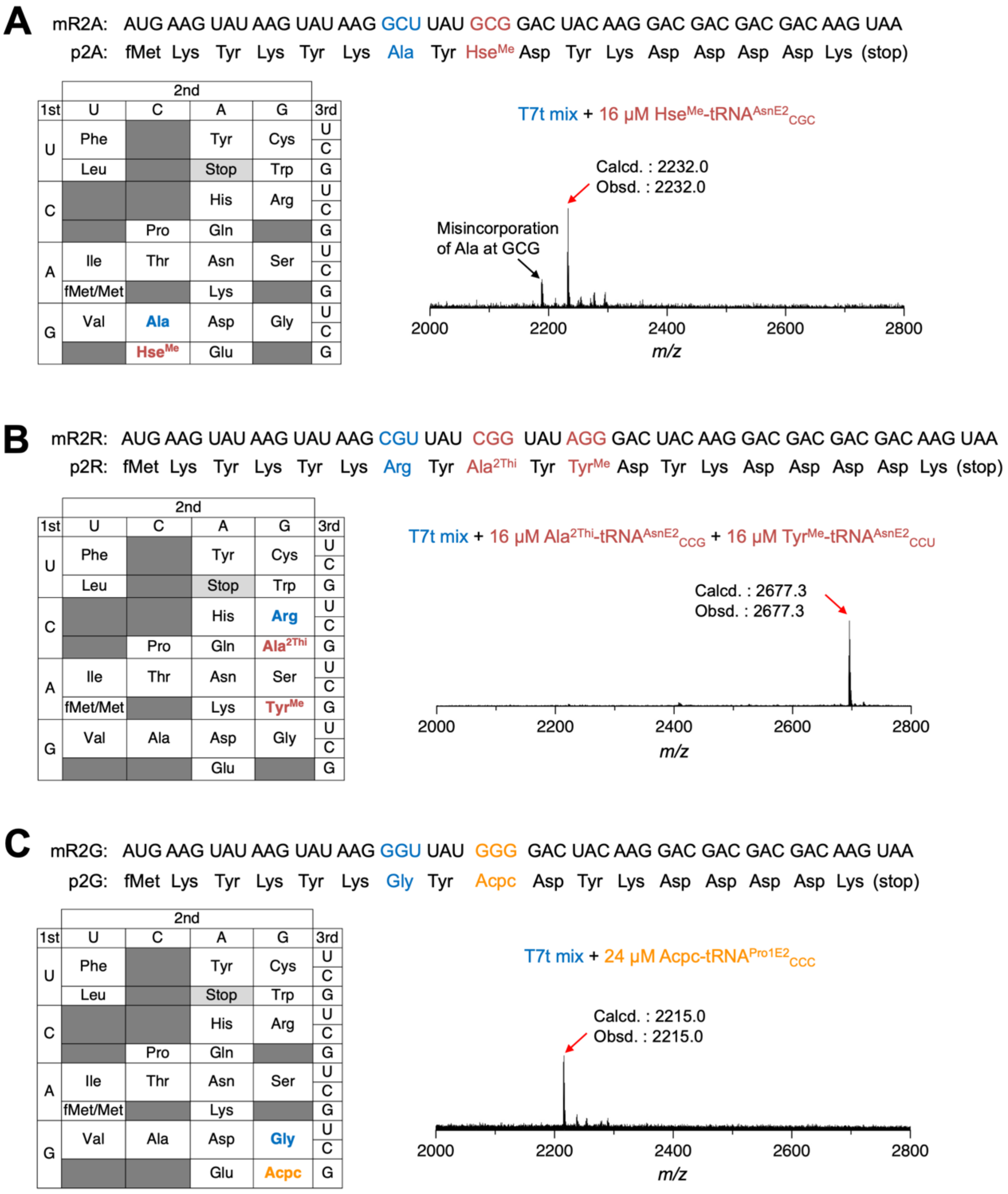

Supplementary Figure 3. Translation of model peptides confirming accurate codon box division. (A−G) Presented are the sequences of the model mRNAs and peptides, the reprogrammed codon table utilized for translation, and the corresponding MALDI-TOF MS spectra of the synthesized peptides. Calculated (Calcd.) and observed (Obsd.) *m/z* values are indicated for each peptide.

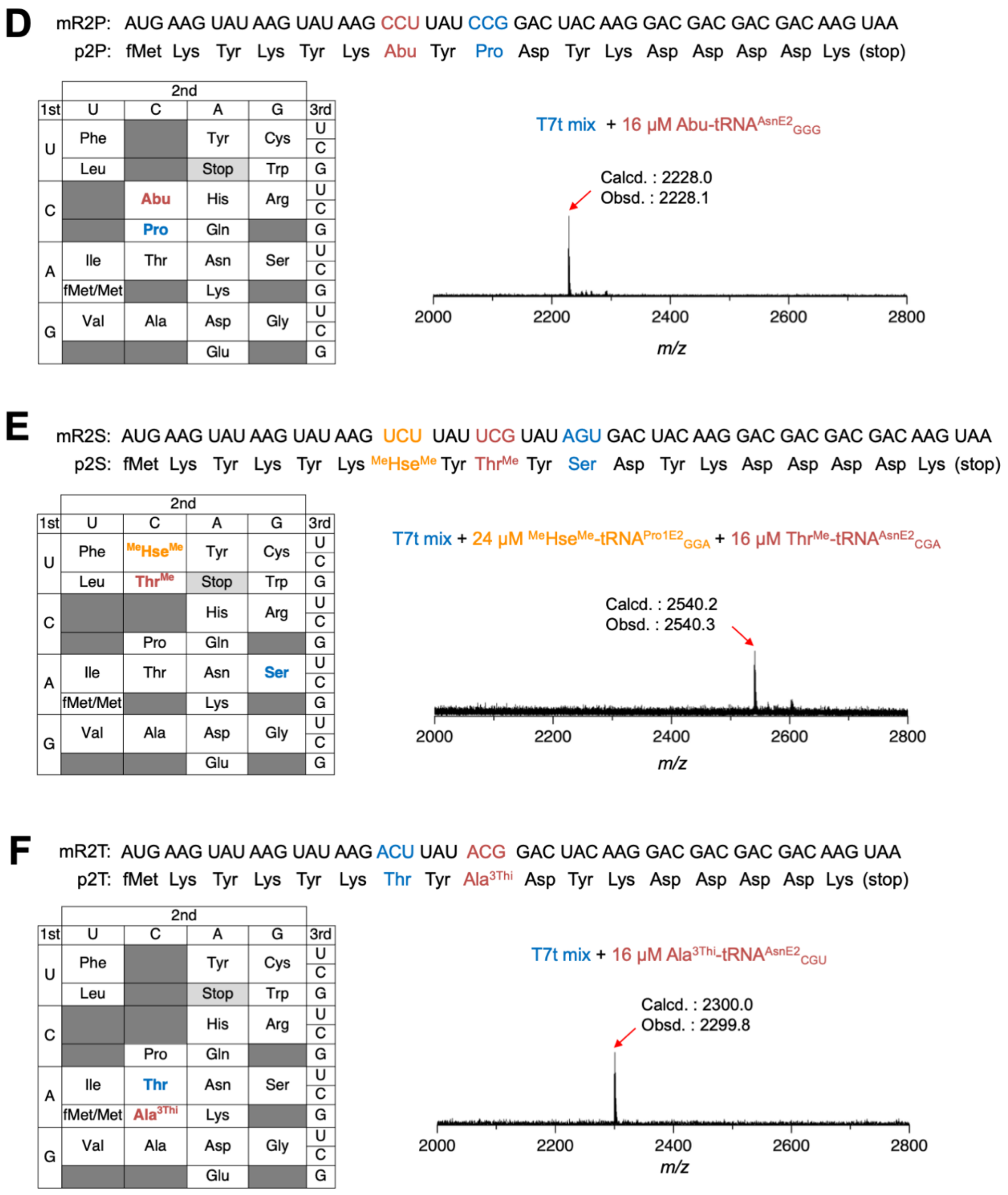

Supplementary Figure 3 continued.

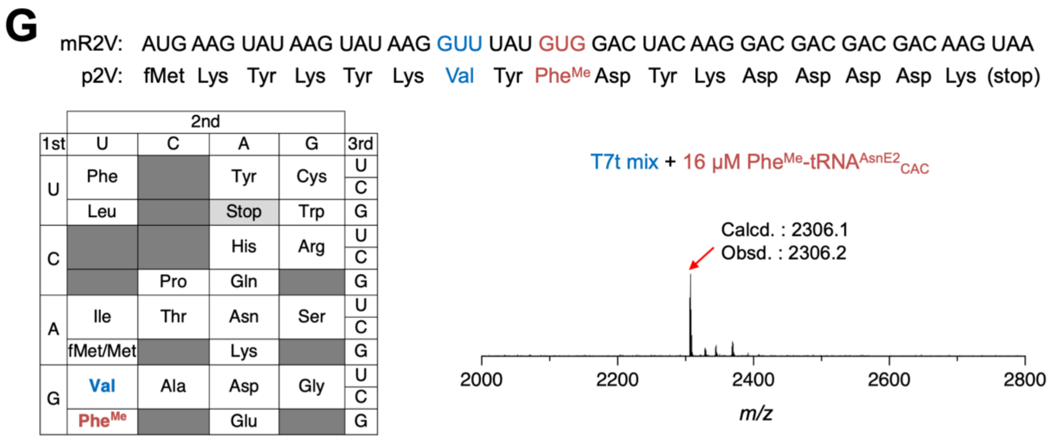

Supplementary Figure 3 continued.

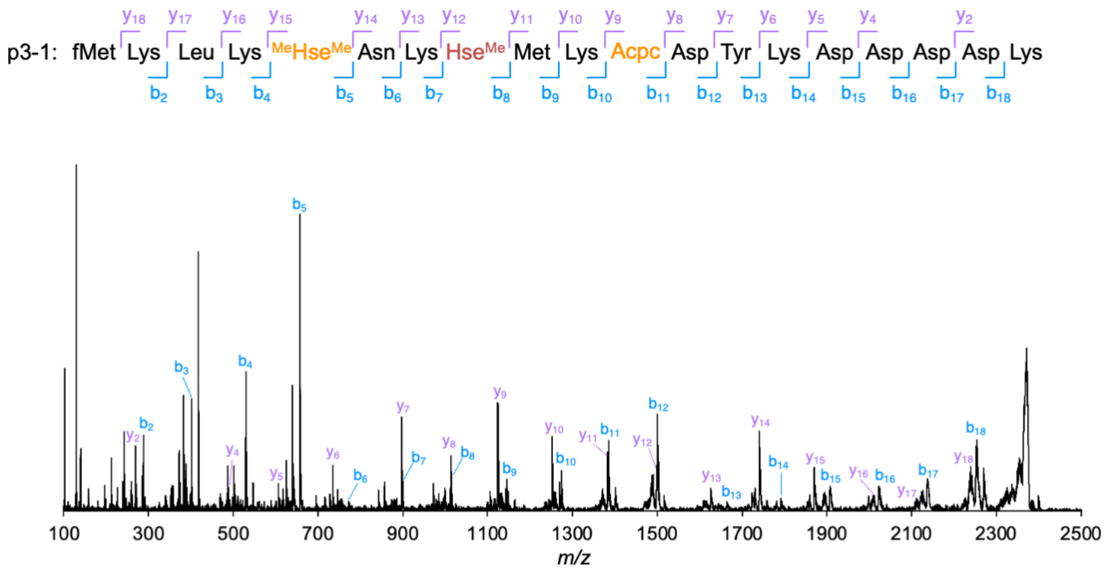

Supplementary Figure 4. ***De novo*** **sequencing of peptide p3-1 by MALDI-TOF/TOF MS/MS.** The observed b and y ions are indicated in blue and purple, respectively.

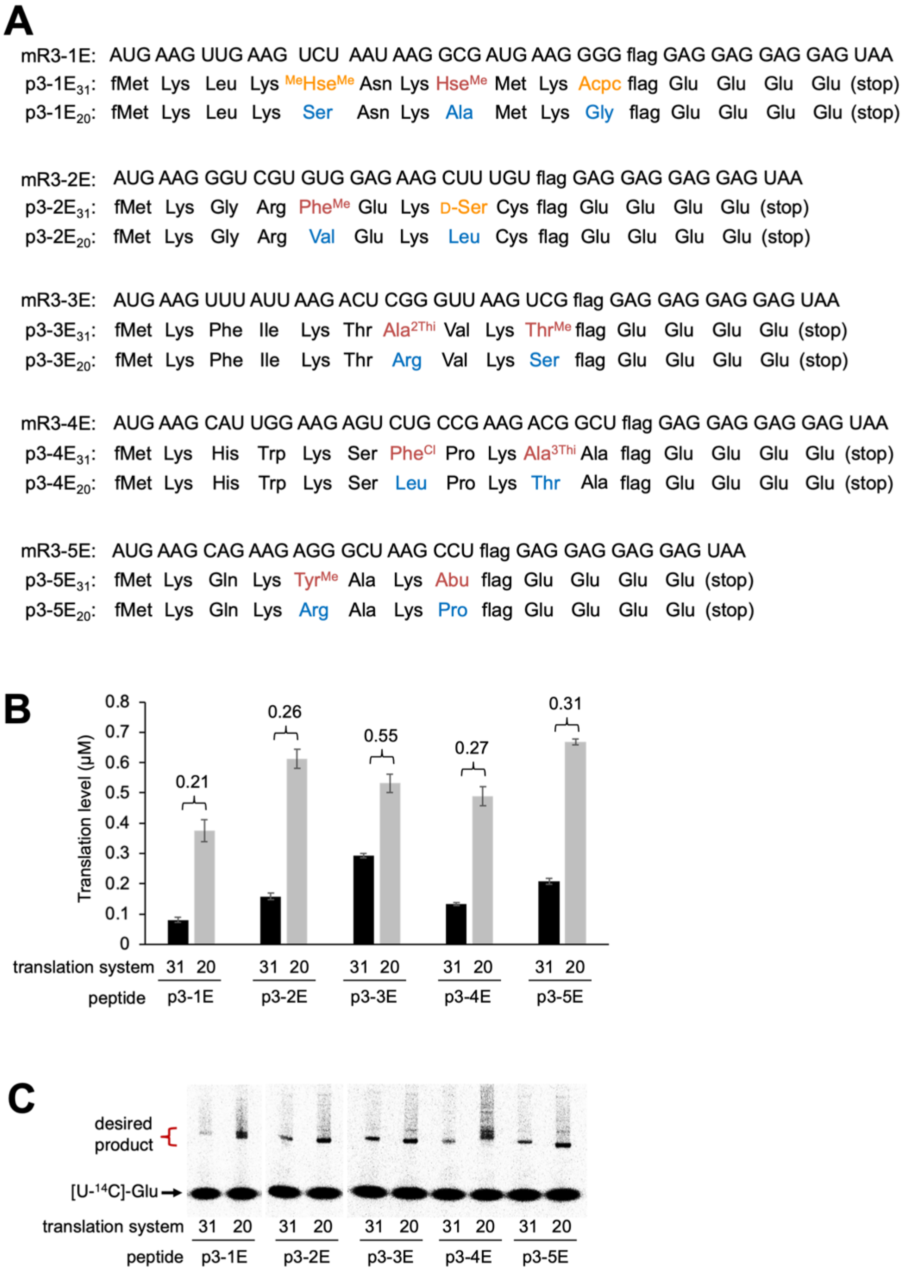

Supplementary Figure 5. Quantification of peptide translation level by radiolabeling. (A) Sequences of model mRNAs and peptides. Translation was performed using either the reprogrammed 31AA system or the canonical 20AA system, yielding the peptides denoted by the subscripts "31" or "20", respectively. Peptides were radiolabeled by [U-^{14}C]-Glu included in the translation mixtures. The RNA and peptide sequences for the "FLAG" tag are 5′-GAC-UAC-AAG-GAC-GAC-GAC-GAC-AAG-3′ and Asp-Tyr-Lys-Asp-Asp-Asp-Asp-Lys, respectively. **(B)** Quantification of model peptide translation levels. Values above the bars indicate the relative translation efficiency of the 31AA system compared to the 20AA system. Error bars represent standard deviations from three independent experiments. **(C)** Tricine SDS-PAGE analysis of model peptide translation. Representative autoradiograms of the SDS-PAGE results are shown; quantitative data derived from these gels are presented in (B).
